# Evaluating the impact of marketing interventions on sugar-free and sugar-sweetened soft drink sales and sugar purchases in a fast-food restaurant setting

**DOI:** 10.1186/s12889-023-16395-z

**Published:** 2023-08-18

**Authors:** Aila Khan, Anna Uro Evangelista, Maria Estela Varua

**Affiliations:** 1 School of Business, Hospitality, Marketing and Sport, Western Sydney University, Sydney, Australia; 2 School of Business, Economics, Finance and Property, Western Sydney University, Sydney, Australia

**Keywords:** Sugar purchases, Sugar-sweetened beverages, Natural experiments, Marketing interventions, Interrupted time series analysis

## Abstract

**Background:**

Beverages high in added sugar, such as sugar-sweetened soft drinks, continue to be associated with various health issues. This study examines the effects of a manufacturer-initiated multicomponent intervention on the sales of sugar-free (SFD) and sugar-sweetened (SSD) soft drinks and the amount of sugar people purchase from soft drinks in a fast-food restaurant setting.

**Methods:**

A database of monthly sales data of soft drinks from January 2016 to December 2018 was obtained from three treatment and three control fast-food restaurants. A multicomponent intervention consisting of free coupons, point-of-purchase displays, a menu board, and two sugar-free replacements for sugar-sweetened soft drinks was introduced in August 2018 for five months in Western Sydney, Australia. A retrospective interrupted time series analysis was used to model the data and examine the effects of the interventions on SFD and SSD sales and their consequential impact on sugar purchases from soft drinks. The analyses were carried out for volume sales in litres and sugar in grams per millilitre of soft drinks sales. A comparison of these measures within the treatment site (pre- and post-intervention) and between sites (treatment and control) was conducted.

**Results:**

The interventions had a statistically significant impact on SFDs but not SSDs. On average, SFD sales in the treatment site were 56.75% higher than in the control site. Although SSD sales were lower in the treatment site, the difference with the control site was not statistically significant. The net reduction of 6.34% in the amount of sugar purchased from soft drinks between sites during the experimental period was attributed to the interventions.

**Conclusions:**

The interventions significantly increased SFD sales and reduced sugar purchases in the short run. Aside from free coupons, the findings support the recommendation for fast food restaurants to nudge customers towards choosing SFDs through point-of-purchase displays and the replacement of popular SSDs with their SFD counterparts.

**Supplementary Information:**

The online version contains supplementary material available at 10.1186/s12889-023-16395-z.

## Background

Sugar-sweetened beverages (SSBs) are consumed daily on a global scale. Research shows that drinks with added sugars provide excess kilojoules with little nutritional value [1]. They also increase the risks of excessive weight gain, dental decay, diabetes, and other chronic conditions [2, 3]. It is estimated that worldwide, diseases associated with sugar-sweetened beverage consumption account for about 184,000 deaths annually [4].

SSB intake levels differ significantly across regions and countries around the world. For instance, Latin America and the Caribbean have the highest median intake of almost four times that in Sub-Saharan Africa and six times the lowest intake region, Asia [5]. In the USA, it is estimated that approximately one-half of adults consume at least one SSB per day [6], while in Australia, 9.1% of adults aged 18 and over consume SSB daily [7]. In the EU, the average proportion of people aged 15 and above who consumed at least one sugar-sweetened beverage per day in 2019 was estimated at around 9%, with Belgium reporting the highest at 20% and Estonia the lowest at 2% [8]. Although Australia appears to consume less than the USA and is in a similar position as the EU on average, it is estimated that more than half of Australians exceed the World Health Organization's (WHO) recommendation to limit free sugars to 10% of daily total energy intake [2]. In Europe, the intake among adults ranges from 7–8% in countries like Hungary and Norway and 16%-17% in countries like Spain and the UK [9]. Despite the extensive literature in the mass media and scientific sources about the potential ill effects of sugar on people's health, consumption of sugar-sweetened beverages in Australia, as in other countries such as the USA, the UK, and Norway, remains high [7, 10].

Reducing the consumption of free sugars (i.e., added sugars) such as those found in SSB is a global public health priority [10]. Research in this area has covered a broad range of interventions such as nutrition labelling, price increases, healthier default beverages, in-store promotion in supermarkets, taxation and other tools designed to alter the environment in which beverage choices are made [11]. Some examples of previous SSB-specific studies include online experiments involving warning labels [12], packaging, taxes in New Zealand [13] and emotional appeals in the US [14]. Researchers have also examined the impact of taxation on SSB sales [15], the effect of a two-pence price levy and other promotional tools on non-alcoholic beverage sales in the UK [16].

A Cochrane review has found environmental interventions to be effective in helping people consume less SSB, that such measures may be used more widely by the government, health professionals, and businesses, and that more research should be undertaken to find out about their short and long-run effects [11]. While the involvement of manufacturers and retailers in sugar-sweetened beverage research is acknowledged, a systematic review of published research conducted by Litman et al. [17] found that industry involvement has been declining significantly with time.

Modifying the in-store environment to incentivise change in purchasing behaviour has been gaining attention in public health research and policy [18–20]. Examples of in-store interventions found to be effective include merchandising or commercial activities aimed to stimulate sales as soon as customers enter the store [21], aesthetic display and signage, placing at proximity to customers at checkout, display in high foot traffic for healthy foods and beverages [22] and monetary incentives to purchase healthier food options in supermarkets [19]. A 2019 Cochrane review found that micro changes in product offerings, such as increasing or decreasing healthy or unhealthy choices, can affect product selection and consumption [23]. A recent systematic review of intervention studies found three behaviour change techniques (BCT) effective in changing purchasing behaviour of food and drinks in supermarkets: prompts/cues, material incentives, and material rewards [24].

Researchers in various areas, including public health, have simultaneously adopted two or more interventions, otherwise known as a multicomponent intervention. Previous multicomponent interventions aimed at reducing the consumption of SSBs focused on integrating various activities around the school curriculum, information, and physical activity sessions for participants. Some studies also looked at media use for targeted messaging [25]. Surprisingly, only a few, except Schwartz et al. [26], have adopted the multicomponent intervention approach [27] using promotion and marketing tactics. Integration of a range of elements of a marketing campaign (e.g., discounts, special offers, advertising) is critical in initiating and sustaining steps in the consumer's 'response' process [28]. This approach is especially relevant in business since most marketing strategies are implemented as an integrated campaign.

The current study adopted a multicomponent approach consisting of merchandising and pseudo-price interventions in a quasi-field experiment designed to increase sales of sugar-free soft drinks and, thus reduce the purchase of soft drinks with added sugars in a fast-food restaurant setting in Sydney, Australia. Because the consumption of soft drinks high in free sugar is commonly associated with fast food restaurants [29], they provide a prime setting to introduce change designed to increase the purchase of sugar-free soft drinks. The study is a response to the call for more research measuring the effects of environmental interventions on sugar consumption [11]. Second, the quasi-field experiment was conducted by a global soft-drink manufacturer, thereby addressing the declining trend in industry involvement [17]. A third feature is that the experiment was conducted in a fast-food restaurant setting, which will not only add to the evidence obtained from supermarkets and grocery stores but will also be replicable in fast-food restaurants in other countries.

## Methods

### Aims

The current study aims to evaluate the effects of marketing interventions on the sales of sugar-free and sugar-sweetened soft drinks and their consequential impact on the amount of sugar people purchase from soft drinks in a fast-food restaurant setting.

### Design and settings

In cooperation with six fast-food chain restaurants, a major global beverage company conducted a quasi-field experiment in two suburban locations in Sydney, Australia. It was a quasi-field experiment because randomisation was not applied to assigning three restaurants to the test and control groups. Instead, the allocation was based on the willingness of the store managers to implement the interventions on the designated dates. An experiment was deemed appropriate, considering the aim was to investigate a causal relationship.

The field experiment was conducted in two suburbs, one designated as the treatment or test and the other as the control site. Both suburbs are in the Western Sydney region and have a similar demographic profile regarding population size, household size, age distribution, and employment levels. Moreover, the two suburbs comprise a mix of ethnicities, with a significantly high percentage of residents speaking a language other than English at home. Very importantly, the prevalence of diabetes in the two suburbs is higher than the national average [30].

### Interventions

Three fast-food restaurants on the test site promoted sugar-free soft drinks over five months as part of their product offerings. A multicomponent approach consisting of five interventions was adopted. As shown in Table 1, five were implemented in the first month, four in the second, and two in the subsequent three months.

**Table 1:** Schedule of marketing interventions

Interventions	Month/Year
1. Coupons of sugar-free soft drinks redeemable in-store (FC)	August 2018
2. Point of purchase kiosk disruptors, window decals, and tray liners, all featuring sugar-free soft drinks (POP)	August – September 2018
3. Replacement of a sugar-sweetened frozen variant with the frozen sugar-free counterpart in the menu (PR1)	August – September 2018
4. Sugar-free soft drinks on menu boards (MB)	August – December 2018
5. Replacement of a sugar-sweetened variant with the sugar-free counterpart in the menu (PR2)	August – December 2018

Below is a description of each:

1. Free coupons (FC) for one major brand (A) and one minor brand (B) of sugar-free soft drinks, redeemable from the test restaurants, were distributed through the local community newspapers and in-store.  This intervention was designed to create further awareness, promote SFDs to the community, and incentivise the public to visit the test restaurants to claim their free SFD.
2. Point of purchase (POP) displays consisting of the following:
  • Window decals (attractive stickers) featuring sugar-free soft drinks were glued to glass windows to attract customers' attention as they approached or entered the restaurant.
  • Kiosk disruptors are computerised stands where customers can view the menu and key in their orders.  During the experiment, the kiosk disruptors displayed banner ads of SFDs only and none of the sugar-sweetened drinks.
  • Colourful paper tray liners featuring SFDs only.
3. Product replacement 1 (PR1) - Brand C's sugar-sweetened frozen drink option was withdrawn from sale and replaced by its SFD counterpart for the duration of the experiment.
4. Menu Board (MB) - Only SFDs were displayed on the menu board during the experiment.  Menu boards with information, images, and prices of SFDs are used to capture attention and remind and influence customers to order SFDs.
5. Product replacement 2 (PR2) – The sugar-sweetened option of Brand B was withdrawn from sale and replaced by its SFD counterpart.

Intervention 1 (FC) promoted the SFD option of two soft drink brands designed to enhance general awareness and invite the public to trial or sample SFDs for free. Interventions 2 and 4 (POP, MB) were used to inform, remind and persuade customers to choose and buy SFDs at the point of sale. The aim of interventions 3 and 5 (PR1 and PR2) was to limit customers' choice [31] to SFDs only.

### Procedure

During the field experiment, all three test restaurants operated as usual. Prior arrangements were made for the promotional materials to be developed and installed on designated dates. The replacement of the sugar-sweetened brands with their sugar-free counterparts was undertaken in coordination with the supplier. The sugar-sweetened drinks were temporarily withdrawn from sale, i.e., unavailable to customers during the designated periods. The frontline staff were unaware that the experiment was taking place. Prior arrangements and coordination among the soft drink supplier, logistics, restaurant managers, and marketing staff contributed to the field experiment's smooth implementation. On the other hand, the control restaurant staff were unaware of the field experiment, except for their senior management.

### Participants

All six restaurants (three tests and three control) operated under the same express service umbrella brand name and had the same product range. During the experiment, it was safe to assume that due to their physical location, all six were exposed to similar extraneous factors such as weather, season and events that could impact sales. The only difference between the two groups of stores was the introduction of the interventions at the test site. Sales reporting during the field experiment for all restaurants proceeded as usual.

### Statistical analysis

A database of monthly sales of soft drinks from January 2016 to December 2018, including the quasi-experiment period (August -December 2018), was obtained from the participating restaurants. A retrospective interrupted time series analysis was used to model the data and examine the effects of the interventions on sales of sugar-free and sugar-sweetened soft drinks and their consequential impact on sugar purchases from soft drinks.

The sugar content of each soft drink item was derived from the nutritional information provided by the manufacturer. All items with no sugar sweeteners were classified as "sugar-free" soft drinks (SFD) and those with sugar sweeteners as "sugar-sweetened "soft drinks (SSD). The monthly sugar content sold in each restaurant was estimated by multiplying each sugar-sweetened soft drink item's sales volume with its sugar content and then aggregated to arrive at each restaurant's monthly total. This monthly total was then divided by the combined sales volume of sugar-free and sugar-sweetened soft drinks to arrive at the associated sugar content in grams per ml of soft drinks sold. The results for all three restaurants in the treatment group were aggregated, and likewise for the control group. As the proportion of sugar-free soft drink sales relative to sugar-sweetened soft drinks increased, the sugar content per ml of soft drinks sold or purchased in that restaurant would decrease and vice versa.

### Interrupted time series analysis

**Fig. 1:**
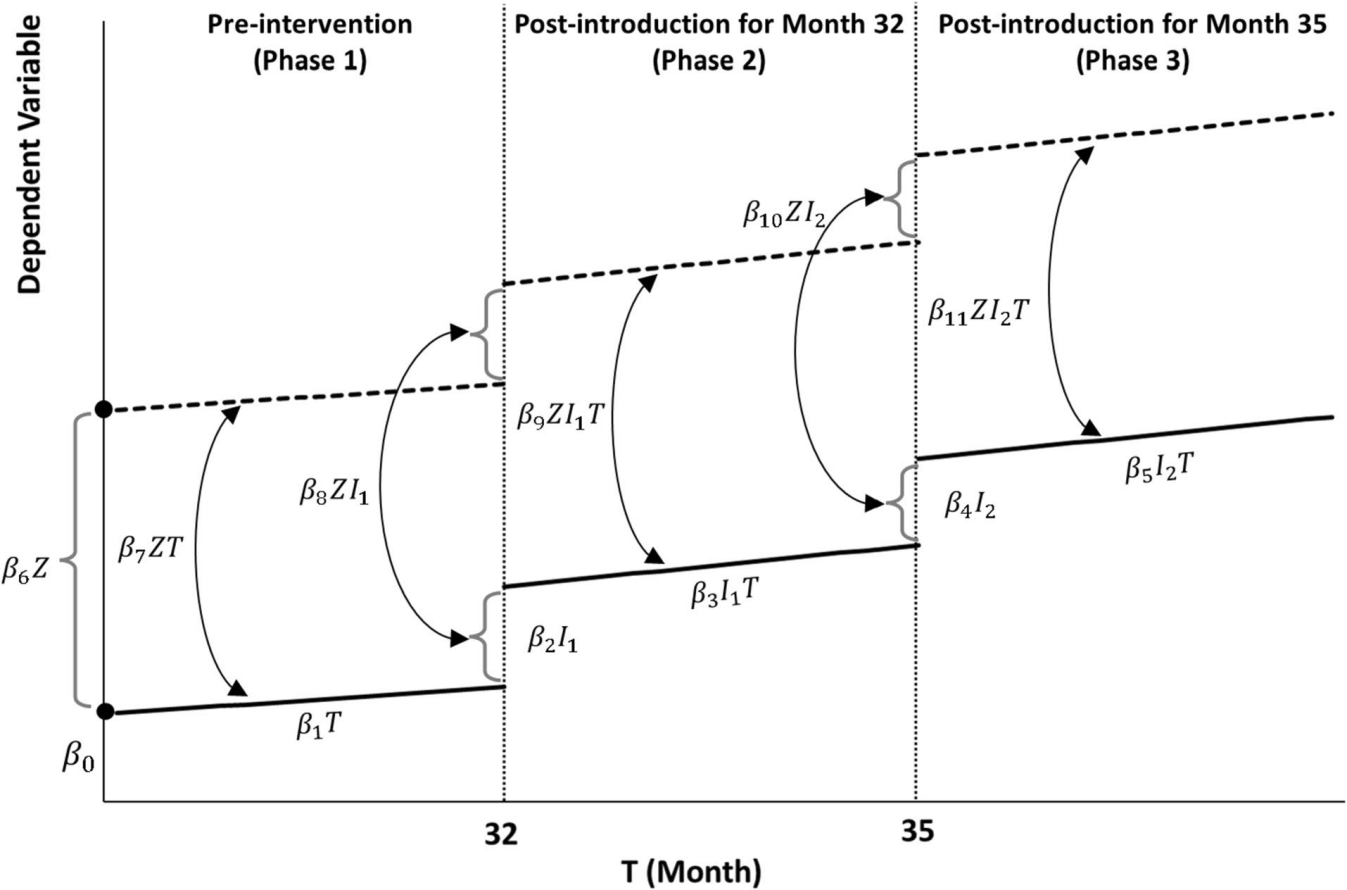
Phases and components of the interrupted time series multigroup analysis. Adapted from Linden [33]

Interrupted Time Series Analysis (ITSA) was adopted as it is "the strongest of the quasi-experimental designs and a powerful tool used for evaluating the impact of interventions" [32 p. 411]. ITSA utilises blocks of time rather than previous values [32]. Each block represents different stages in the experiment: before, at the time of introduction and post-introduction, as shown in Fig. 1.

The method initially assumes that the data changes gradually over the entire series. Any abrupt change from the baseline trend could only be due to the intervention [33]. The observations before the interventions was used to establish the baseline trend. All models were estimated using Prais-Winsten regression. The coefficients are less biased as the software adjusted the estimates for any existing serial correlation of the order AR(1). The analysis was carried out in two phases: a single group or within-site (treatment group only) and a multigroup or between sites (between treatment and control). The single group analysis and results are presented in the supplementary section inclusive of Figures C1 to C5, Tables C1, C2 and D1 to D4. Those of the multigroup or between sites are presented in the sections that follow.

The equation for the entire multigroup analysis is shown below

1 $${Y}_{t}= {\beta }_{0}+ {\beta }_{1}{T}_{t}+ {\beta }_{2}{I}_{{1}_{t}}+ {\beta }_{3}{I}_{{1}_{t}}{T}_{t}+ {\beta }_{4}{I}_{{2}_{t}}+ {\beta }_{5}{I}_{{2}_{t}}{T}_{t}+ {\beta }_{6}{Z}_{t}+ {\beta }_{7}{Z}_{t}{T}_{t} + {\beta }_{8}{Z}_{t}{I}_{{1}_{t}}+ {\beta }_{9}{Z}_{t}{I}_{{1}_{t}}{T}_{t}+ {\beta }_{10} {Z}_{t}{I}_{{2}_{t}}+ {\beta }_{11}{Z}_{t}{I}_{{2}_{t}}{T}_{t}+{\epsilon }_{t}$$

where:

*T*_*t*_ – month (1 to 36)

*Z*_*t*_ – site (= 1 for treatment site, = 0 for control site)

*I*_1*t*_ – introduction of all interventions before or during the month (= 1 for months 32 to 36, = 0 for other months)

*I*_1*t*_*T*_t_ – post-introduction phase after all interventions were introduced

*I*_2t_ – retention of interventions 4 & 5 (= 1 for months 35 & 36, = 0 for others)

*I*_2t_*T*_*t*_ – post-introduction phase after retaining interventions 4 & 5

The equations above were estimated separately for volume sales of sugar-free drinks, sugar-sweetened drinks, the sugar content of soft drinks sales, and the corresponding monthly proportion change. Sugar content was calculated based on grams per ml for each soft drink variant and the volume sold. The estimated models are shown in Supplementary Table A1. The autocorrelation in the SSD sales and sugar purchase models was addressed by adding a lag term (${y}_{t-1}$) to account for the carry-over effects from previous months but could not be completely corrected. It was inconclusive at 5% for both models. The proportion change in sugar content was calculated and modelled. The resulting observations took into account of carry-over effects.

The equations for the control site and the treatment site are shown below after substituting the relevant value for ${Z}_{t}$. The model estimates for these equations are shown in Supplementary Tables A1 and A2.

Control site ($${Z}_{t}=0$$)

2 $${Y}_{t}= {\beta }_{0}+ {\beta }_{1}{T}_{t}+ {\beta }_{2}{I}_{{1}_{t}}+ {\beta }_{3}{I}_{{1}_{t}}{T}_{t}+ {\beta }_{4}{I}_{{2}_{t}}+ {\beta }_{5}{I}_{{2}_{t}}{T}_{t}+{\epsilon }_{t}$$

Treatment site ($${Z}_{t}=1$$)

3 $${Y}_{t}= {(\beta }_{0} + {\beta }_{6})+\left({\beta }_{1}+{\beta }_{7}\right){T}_{t}+\left({\beta }_{2}+{\beta }_{8}\right) {I}_{{1}_{t}}+\left({\beta }_{3}+ {\beta }_{9}\right){I}_{{1}_{t}}{T}_{t}+ \left({\beta }_{4}+ {\beta }_{10}\right){I}_{{2}_{t}}+\left({\beta }_{5}+ {\beta }_{11}\right){I}_{{2}_{t}}{T}_{t}+{\epsilon }_{t}$$

## Results

Overall, the multicomponent intervention effectively reduced the amount of sugar associated with soft drink purchases. Compared to the pre-intervention period, sugar purchases dropped by 4.87% per month in the treatment site while an increase of 1.47% was recorded in the control site. The net reduction of 6.34 percentage points based on the interrupted time series analysis was attributed to the interventions. During the experimental period, the actual average monthly sugar purchases in the treatment site were 7.18% lower than in the control site. Throughout the experiment, the reduction in sugar purchases was greater when there were more rather than fewer active interventions. A comparison of the results of each phase of the experiment against the pre-intervention period, including the five interventions' relative effectiveness, is presented in the following sections.

### Changes in sugar-free soft drink sales

The most significant impact of the interventions was on SFD sales. As shown in Table 2, SFD sales were significantly higher in the treatment site compared to the pre-intervention period, with the highest net increase recorded in month 32 when all five interventions were active. SFD sales in the treatment site were, at the least, double the monthly pre-intervention average, while the control site recorded only a modest rise of between 17.65% and 51.53%. The statistical significance of this difference is shown in Supplementary Table A1.

**Table 2:** Comparison of volume sales for sugar-free drinks in the intervention phases against the pre-intervention period

Month (Phases)	No. of Interventions	Against pre-intervention			Intervention Effect
		**Treatment**		**Control**	**Percentage Points**
32	5	274.55%	17.65%		256.90%
33–34	4	221.11%	37.73%		183.38%
35	2	102.69%	18.10%		84.59%
36	2	107.38%	51.53%		55.85%

^*^Monthly average

**Fig. 2:**
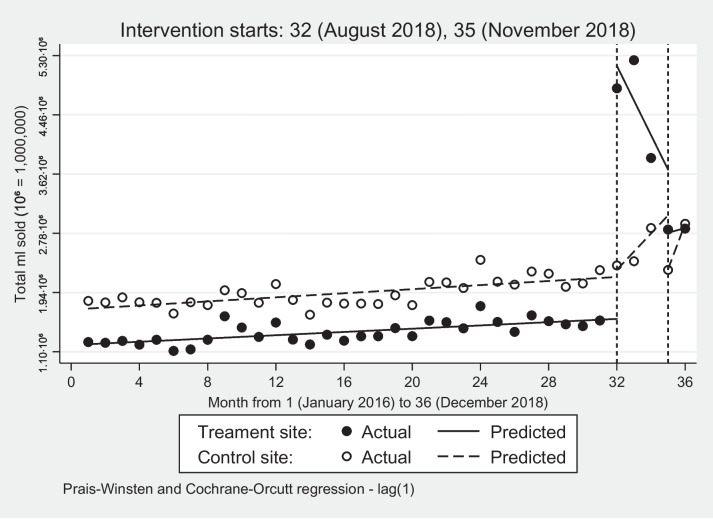
Volume sales of sugar-free drinks: treatment and control sites

Figure 2 shows that the interventions disrupted the sales trend at the treatment site. At pre-intervention (month 1–31), SFD sales in both sites had a similar upward trend, with the former exhibiting a slightly higher steady increase. When the interventions were introduced in month 32, the treatment site registered a statistically significant greater increase (231.44%) than the control site (5.39%).

When the free coupons were withdrawn in months 33–34, SFD sales in the treatment site decreased by an average of 10.01%, while the control site had an average increase of 10.79%. This statistically significant difference indicates the sales effects of the free coupons (FC) (Supplementary Table A1). Likewise, the withdrawal of interventions 2 and 3 (POP PR1) in month 35 led to a similar result, with sales declining by 33.17% against a drop of only 18.22% in the control site. The growth in volume sales after that (month 36) was insignificant in both sites as shown in Table 3. The treatment site had 56.75% more volume sales than the control due to the interventions (Supplementary Table B1).

**Table 3:** Linear trend post-introduction: treatment and control sites

		Sugar-free drinks (Total ml)	Sugar-sweetened drinks (Total ml)	Sugar content sales (Grams per ml)	Proportion Change^2^ Sugar content sales
Month 32	Treatment site	-490,000.00^a^	5,858,613.00	0.0040^a^	0.0692^a^
		(111,000.00)	(3,310,000.00)	(0.0010)	(0.0115)
	Control site	257,000.00^b^	5,763,805.00	-0.0013	-0.0141
		(111,000.00)	(3,300,000.00)	(0.0009)	(0.0115)
	Difference	-748,000.00^a^	94,807.95	0.0053^a^	0.0834^a^
		(156,000.00)	(4,670,000.00)	(0.0013)	(0.0162)
Month 35	Treatment site	64,600.00	15,131,195.00^b^	0.0013	0.0097
		(238,000.00)	(6,030,000.00)	(0.0014)	(0.0196)
	Control site	643,000.00^a^	19,230,158.00^a^	-0.0011	-0.0135
		(238,000.00)	(6,060,000.00)	(0.0014)	(0.0196)
	Difference	-578,000.00	-4,098,963.00	0.0024	0.0233
		(337,000.00)	(8,370,000.00)	(0.0020)	(0.0277)

Legend: ^a^ (Significant at 1% & 5%); ^b^(Significant at 5% only)

1. The monthly change is: $\Delta = {SugarContent}_{t}- {SugarContent}_{t-1}$ where t is the current month, and t-1 is the previous month

2. The proportion change is: $proportion change= \frac{{SugarContent}_{t}- {SugarContent}_{t-1}}{{SugarContent}_{t-1}}$

### Changes in sugar-sweetened soft drink sales

The interventions had a minimal impact on SSD sales. Compared to the preintervention period, SSD sales declined in both sites (Table 4). However, despite the relatively larger decline in the treatment site as expected, it was not substantive enough to register a statistically significant outcome.

**Table 4:** Comparison of volume sales for sugar-sweetened drinks in the intervention phases against the pre-intervention period

Month (Phases)	No. of Interventions	Against pre-intervention		Intervention Effect
		Treatment	Control﻿	Percentage Points
32	5	-12.66%	-11.60%	-1.06%
33–34	4	-21.66%	-15.07%	-6.59%
35	2	-14.09%	-4.60%	-9.49%
36	2	2.75%	10.77%	-8.02%

^*^Monthly average

**Fig. 3:**
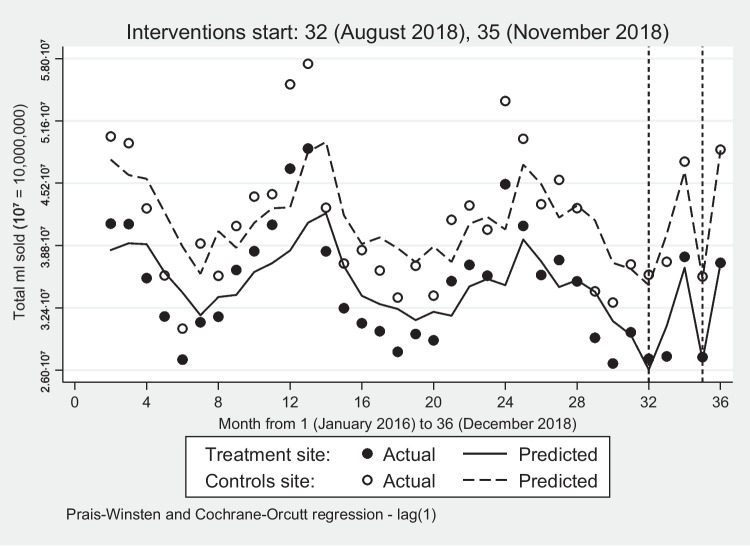
Volume sales of sugar-sweetened drinks: treatment and control sites

During the pre-intervention period, SSD sales in both sites gradually decreased, but unlike SFDs, SSDs seem to follow a seasonal pattern with apparent peaks and troughs at almost regular intervals (Fig. 3). In month 32, when all five interventions were introduced, SSD sales fell by 12.05% in the treatment and 4.61% in the control site which, like in the remaining months, were not statistically significant. It is likely that as SSD sales have historically been huge, these declines were not substantive enough to register a statistically significant outcome. It is also possible that SSB sales in the control site were more volatile (Fig. 3), thus, more sensitive to small changes. The equivalent volume amounts are calculated in Supplementary Table B2.

### Changes in sugar purchases

The impact of the interventions on sugar purchases, i.e. the amount of sugar people purchased from soft drinks, which is the focus of the study, has been found to be statistically significant. A negative change which was the interventions' objective, was consistently recorded in the treatment site. The highest net effect, i.e. treatment less control, was recorded at introduction (month 32) when all five interventions were active. Throughout the experiment, the drop in total sugar purchases was greater when there were more active interventions than fewer. The number of active interventions had a significant impact. The detailed calculations are in Supplementary Table B3.

Table 5 shows that sugar purchases were significantly reduced by up to 6.6% in the treatment site compared to a positive increase of up to 4.17% in the control site. Overall, an intervention effect of -10.77% was achieved when all five interventions were offered. The relative effectiveness of each intervention is discussed in a separate section. Thus, despite the failure to significantly disrupt SSB sales, the interventions have caused a significant reduction in the amount of sugar purchased from soft drinks.

**Table 5:** Comparison of the sugar content of soft drink sales in the intervention phases against the pre-intervention period

Month (Phases)	No. of Interventions	Against pre-intervention		Intervention Effect	
		**Treatment**	**Control**	**Percentage Points**	**gms per ml**
32	5	-6.60%	4.17%	-10.77%	-0.01025
33–34	4	-6.46%	1.60%	-8.06%	-0.00789
35	2	-3.45%	0.61%	-4.06%	-0.00443
36	2	-1.38%	-0.65%	-0.73%	-0.00155

^*^Monthly average

**Fig. 4:**
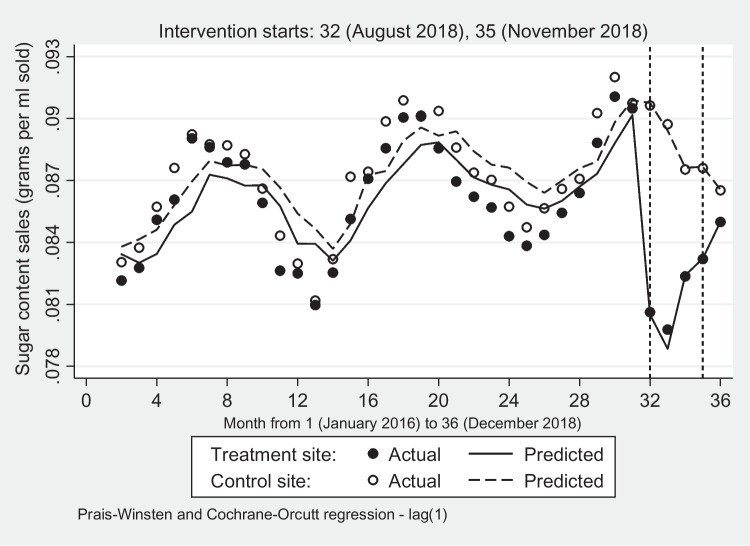
Sugar content of soft drink sales: treatment and control sites

Figure 4 shows that during the pre-intervention period (months 1–31), sugar purchases in both sites were increasing with regular peaks and troughs, similar to SSD sales, although less volatile. The five interventions in month 32 successfully disrupted the rising trend in the treatment site, dropping sharply by 10.71% (*p*-value < 0.01) compared to the not significant decrease of 0.14% in the control site. The withdrawal of the free coupons (FC) in months 33 & 34, however, led to an increase in sugar purchases by an average of 1.22% (*p*-value < 0.01), while the opposite occurred in the control site where these continued to drop by 1.74% possibly due to the carry-over effects from previous months. A further reduction in the number of interventions led to an increase in sugar purchases in the treatment site brought about by a bigger drop in SFD (-33.17%) than in SSB (-25.98%) sales, while the control site saw only a small, not significant increase of 0.04%. Despite the rise in sugar purchases in month 35, these were still lower than those in the pre-intervention phase (Table 5).

### Proportion change in sugar purchases

Proportion change refers to the change in the amount of sugar purchased from soft drinks in the current month relative to that of the previous month, expressed in percentages. The detailed calculations are shown in Supplementary Table B4. The estimated proportion change model forms the basis for estimating the amount of sugar (in gms) associated with one ml of soft drinks sales or sugar purchased from soft drinks. A comparison of sugar purchases during the experiment against the pre-intervention period (Table 6) shows that the interventions had a significant impact.

**Table 6:** Comparison of the adjusted sugar content of soft drink sales based on proportion change in the intervention phases against the pre-intervention period

Month (Phases)	No. of Interventions	Against pre-intervention		Intervention Effect	
		**Treatment**	**Control**	**Percentage Points**	**gms per ml**
32	5	-6.28%	3.69%	-9.97%	-0.0056
33–34	4	-7.43%	1.59%	-9.03%	-0.0052
35	2	-4.51%	0.56%	-5.06%	-0.0037
36	2	-2.38%	-0.67%	-1.71%	-0.0025

^*^Monthly average

**Fig. 5:**
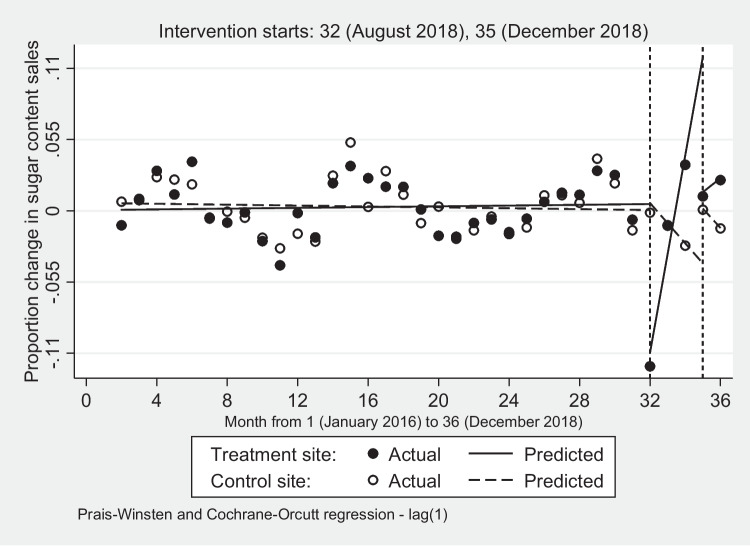
Monthly proportion change in sugar content of soft drink sales: treatment and control sites

Based on Fig. 5 and the estimates in Supplementary Table B4, the rate of change in sugar purchases in the treatment site increased in the pre-introduction period while it decreased in the control site. In month 32, when the five interventions were introduced, the treatment site, as expected, had a drop in sugar purchases, but as the number of interventions decreased, sugar purchases increased sharply up to month 35. All these changes were statistically significant (*p* < 0.01), while the control site's change rates were not.

### Comparison of intervention effects

This section focuses on the relative effectiveness of the five interventions to determine which intervention had a greater or lesser impact on sugar purchases. The evaluation is based on the results in Tables 5 and 6. For a more detailed computation, please refer to Supplementary Tables B3 and B4.

While intervention 1 (FC), as indicated in earlier sections, was found to have statistically significant effects on SFD sales and sugar purchases, the impact of interventions 2 & 3 (POP & PR1) was less evident. As shown in Table 5, intervention 1(FC) which was introduced in month 32, resulted in a difference of 10.77 percentage points in the sugar purchases between treatment and control sites. Interventions 2 & 3 (POP & PR1), which were active in months 33–34, yielded a smaller difference of 8.06 percentage points, with a 6.46% drop in the treatment site, indicating that POP and PR1 were less effective.

The subsequent interventions (MB & PR2; Table 5) led to a relatively smaller difference in sugar purchases between the two sites. Although PR2 involved a product replacement similar to intervention 3, the brands differed. The brand for PR1 was more popular. This result implies that while product replacement could be an effective strategy for reducing sugar purchases, the type or brand of soft drink could also affect the outcome.

The proportion change model further confirms the effectiveness of intervention 1 (FC) and provides more convincing evidence that the succeeding interventions still had a significant role in reducing sugar purchases. As Table 6 shows, from months 33–34, interventions 2 and 3 (POP and PR1) effectively reduced sugar purchases. These findings indicate that POP and PR1 were as effective as FC in reducing the amount of sugar people purchased from soft drinks during the experiment.

## Discussion

Limited evidence has been gathered on the real-world impact of replacing sugar-sweetened drinks (SSDs) with sugar-free drinks (SFDs) over time in community-retail settings. This multicomponent intervention evaluated the effect of industry-initiated marketing interventions on purchasing behaviour over time. By designing immersive experiences that directly engage the user, the interventions used in this study were implemented to uncover whether the marketing interventions can motivate behaviour towards the purchase of SFDs.

Changes in the SFD and SSD sales, sugar purchases, the monthly percentage change in sales, and monthly proportion change in sugar purchases were the outcome measures used in the study. Although the results reveal that the impact of the interventions is consistent and significant for most measures other than SSD sales, the sustained implications are not.

### Impact on customer purchase and amount of sugar purchased from soft drinks

The control and treatment site data results confirm that the interventions' immediate effect was a significant increase in SFD sales and a reduction in sugar purchases (in terms of SSD volume, monthly change and monthly proportion). This is expected as all five interventions were implemented in month 32. Furthermore, there is an incentive for consumers to use the coupons and avail of SFD for free. The greater price differential between SFD (with free coupon) and SSD is the primary motivation for the observed changes in customer purchasing, rather than customer health consciousness per se. The findings of previous studies support that price differential will change consumer purchases or would do so if they had been aware of it [34, 35]. Although the redeemable coupons in month 32 boosted existing consumers' engagement and introduced SFD to new consumers, they only lasted a month.

With four interventions in operation in months 33 and 34, the results indicate that the volume of SFD sales decreased while sugar purchases increased (from the previous month). Since consumers then had to pay for the drink, some would have purchased the type they preferred. It is noted that SSD was available in-store except for Brands B & C, although not listed on the menu board. Previous studies on sugary drinks have shown that taste, convenience, and price rank highly [36]. Furthermore, other studies have reported that the population groups with high sugary drink consumption, namely young adults, males and the most disadvantaged, are more likely to be influenced by the "preferred brand" and taste than ingredients and information [37, 38]. Adolescents reported very strong perceived taste preferences for sugared sodas, and they preferred sugar-sweetened sodas in blinded testing [39]. Moreover, some consumers may have hesitated to switch to SFDs due to negative information regarding SFDs. Some studies indicate that SFDs may cause problems ranging from headaches to an increased risk of developing diabetes [40].

When the number of interventions was reduced to two in months 35 and 36, the result was an increase in SFD sales and a reduction in sugar purchases. The result can be partly explained by chooser and menu-dependent preferences [41]. Chooser dependence means that one's preference might reverse depending on whether a choice is made by oneself or someone else. The reversal may lie in the individual's desire to abide by a social norm. A person who would never choose to take the most significant slice of a cake may be happy to be forced to take it. For those non-regular customers or those consumers who need to be made aware of the availability of SSD in store, they will rely on the information on the menu board and will thus order SFDs. Similarly, menu dependence refers to a preference change caused by an extension or contraction of a choice set. Its reason may again be a social norm. A person choosing not to take the last biscuit may have a different preference if plenty is left over for others. In this field experiment, the treatment sites replaced the sugar-sweetened variant with their sugar-free counterparts in their menus, thus limiting the choices available to consumers. The consumer then wanted to purchase the drink during the designated period and had no alternative but to buy the SFD variants. Noting that the interventions effectively changed the customers' in-store sugar purchase behaviour when given a limited choice set, replacing the sugar-sweetened variant with the sugar-free counterpart on the menu makes sense.

The positive sign of the lag variable may indicate addictive or habitual consumption and, thus, the continued patronage of SSDs. A previous study [39] reported that perceived soda preferences significantly affect soda consumption and that children whose parents were regular soda drinkers were 2.9 times as likely to drink sweetened sodas.

However, it is interesting to note that although the SSD sales were lower in the treatment site, it was not significantly different from the control site. This result can be due to several reasons. One explanation is that buying an SSD in a retail shop is a habit for some consumers. Psychological theories of habit posit that when a strong habit is formed through behavioural repetition, it can trigger behaviour automatically in the same environment. A recent study by Judah et al. [42] found that individuals with a stronger habit of drinking sugary drinks have limited control over their drink behaviour. The same study reported that when exposed to substitute beverages, consumers do appear to be able to take control over their drink behaviour and respond to the intervention accordingly. Then again, the interventions implemented in this study that declined in number and were of short duration were not long enough to change consumer purchase behaviour. Another is explained by 'taste'. A recent study in Australia by Dono et al. [43] indicated that 'taste' was a ubiquitous reason for purchase (94%), followed by 'easily available' (76%) of sugary drinks. Our results support the argument for making SFD the default option in meal deals and adding a health levy to SSDs to increase their price and expand the price differential with SFDs.

#### Long-run implications

Standard economic theory assumes that individual preferences are stable but may be altered by a side condition such as information [44]. Altered behaviour can reliably be traced back to changes in the individual's knowledge of the product. When new information becomes available, individuals may recognise a superior option and change their behaviour. Further information can become available through an exogenous shock and endogenously through learning effects connected to past decisions. For example, with experience goods, consumption is necessary to acquire information about product quality. Thus, the impact of the interventions in this field experiment may take longer to observe (hence a not significant post-intervention result) and may also vary over time depending on how familiar and knowledgeable consumers become with the alternative healthier choice, such as the SFDs.

### Interventions compared

The effects between the pre and post-intervention periods in the treatment and control sites were also estimated further to validate the effectiveness of this study's five interventions. The results confirm that the multicomponent intervention effectively reduced on-site sugar purchases from soft drinks. This analysis shows that point-of-purchase displays and product replacement have a comparable impact as coupons. Evidence supports that point-of-purchase displays could act as triggers [45] or nudge customers [46] to order an SFD instead of an SSD. Nudging at the right time, as when customers place their orders in restaurants, would help customers choose, especially with impulse products such as soft drinks. Considering further that the POP intervention included computerised kiosk disruptors where customers could directly type in their orders, such a device displaying SFD banner ads must have been very attractive to teenagers who frequent fast-food chains. The product replacement intervention, which restricted customers' choice to the SFD option, seems to have worked very well, considering that Brand C is a favourite of young people. Relative to PR2, PR1 was more effective, most likely because Brand C is more popular than Brand B. This implies that while product replacement could be an effective strategy for sugar reduction, it should be used in conjunction with the appropriate brand of soft drinks in a fast-food restaurant setting.

### Conclusions & recommendations

The findings of this study indicate that the interventions applied were significant in increasing SFD sales and reducing on-site sugar consumption (in terms of SSD volume, monthly change and monthly proportion) in the short run but not in the long run (sustained). By designing experiences that directly engage the customers, such as the kiosk disruptors and other point-of-purchase tools, manufacturers and fast-food operators could entice or nudge customers, particularly the younger cohort, to order the SFD variant of their favourite brand of soft drinks. If found enjoyable, the experience in the fast-food chain could have extended effects on subsequent purchases or other occasions of use, such as cinemas, sporting events and at-home consumption. Product replacement, as shown by the results consistent with previous findings about replacing unhealthy options with healthier ones in supermarkets, is also a promising strategy. Considering that one product replacement was more effective than the other in this study, SFT replacement strategies must consider the brand, format (frozen or liquid) and other product attributes for it to work.

Since the reasons for purchasing SSD are associated with numerous factors, multi-level interventions will be required to target sugary drink consumption effectively. This emphasises the need for policymakers, public health advocates and the soft-drink industry to collaborate when planning interventions and policies to implement. This is particularly important in Australia, where there needs to be more policy progress in this area.

The analysis conducted in this study is based on habitual patterns and time series sales data. The method can provide a foundation for better assessing future sugar consumption behaviour. The study also paves the path towards encouraging greater industry involvement in future sugar consumption studies and developing delightful SFD options for sugary drinks.

### Limitations and future research

The variation in the number of interventions during the experiment may partly explain why the sustained effects of the interventions were not achieved. The long-run impact of the field experiment could have been established much better if the number of interventions was kept constant, the sales data were collected more frequently, and the field experiment period was extended. A long time series before, during and after the interventions would provide greater confidence in the reliability of the claimed relationships between the interventions and their outcomes [47]. Future studies should therefore address these issues when planning a field experiment. Careful attention should also be given to the selection of testable interventions. Considering that a multicomponent approach is suited for marketing field experiments, the BCT taxonomy should be used to help identify relevant interventions to reduce sugar consumption or address similar health issues. The study's results allow testing the effects of intervention-initiated SFD purchases in fast food restaurants by extending it to other product use situations such as sporting and community events, concerts, festivals or even in-home consumption.

### Supplementary Information

**Additional file 1: Table A1.** Results of interrupted time series analysis: treatment and control sites.

**Additional file 2: Table A2.** Calculated combined coefficients from the results: treatment and control sites.

**Additional file 3:** Legend for Tables B1 to B4.

**Additional file 4: **Tables B1 to B4.

**Additional file 5:** Explanation for Single Site Treatment Results.

**Additional file 6: Figure C1.** Phases of the single group interrupted time series analysis.

**Additional file 7: Figure C2.** Volume sales of sugar-free drinks: treatment site.

**Additional file 8: Figure C3.** Volume sales of sugar-sweetened drinks: treatment site.

**Additional file 9: Figure C4.** Sugar purchases: treatment site.

**Additional file 10: Figure C5.** Monthly proportion change in sugar consumption: treatment site.

**Additional file 11: Table C1.** Results of the interrupted time series analysis: treatment site.

**Additional file 12: Table C2.** Linear trend post-introduction: treatment site.

**Additional file 13:** Legend for Tables D1 to D4.

**Additional file 14:** Tables D1 to D4.

## Data Availability

The datasets analysed during the current study are not publicly available due to their proprietary nature but may be available from the corresponding author upon reasonable request.

## Abbreviations

AIHW: Australian Institute of Health and Welfare
BCT: Behaviour change techniques
Gms/ml: Grams per millilitre
ITSA: Interrupted time series analysis
Mg/ml: Milligrams per millilitre
NHMRC: National Health and Medical Research Council
SSB: Sugar-sweetened beverage
SSD: Sugar-sweetened soft drinks
SFD: Sugar-free soft drinks
WHO: World Health Organisation
