# Supplementary material for: Evaluating the impact of marketing interventions on sugar-free and sugar-sweetened soft drink sales and sugar purchases in a fast-food restaurant setting
Source: BMC Public Health. 2023 Aug 18;23:1578. doi: 10.1186/s12889-023-16395-z (PMC10439673; doi:10.1186/s12889-023-16395-z)
Supplement: Supplementary file 1 — Additional file 1: Table A1. Results of interrupted time series analysis: treatment and control sites. [file 12889_2023_16395_MOESM1_ESM.docx]

**Table A1 - Results of interrupted time series analysis: treatment and control sites**

|  | Dependent Variable ($y_{t}$) | | | |
| --- | --- | --- | --- | --- |
|  | Sugar-free drinks (Total ml) | Sugar-sweetened drinks  (Total ml) | Sugar purchased  (Grams per ml) | Proportion Change^3^  Sugar purchased |
| Intercept ($\beta_{0}$) | 1,712,962.70^***^ | 20,701,560.00^***^ | 0.03613533^***^ | 0.0052815 |
|  | (48,909.44) | (5,561,935.00) | (0.00824350) | (0.0100800) |
| Time ($\beta_{1}$)  (Month 1 to 36) | 14,469.358^***^ | -35,075.31 | 0.00005548 | -0.00015723 |
|  | (2,805.92) | (134,709.50) | (0.00006360) | (0.00058820) |
| Month 32 ($\beta_{2}$)  (1-Month 32, 0-Other) | 1,012,38.33 | -3,629,065.60 | 0.00054436 | 0.00490275 |
|  | (148,790.30) | (4,917,793.00) | (0.00136380) | (0.01760640) |
| Month 32 $\times$ Time ($\beta_{3}$) | 242,900.53^***^ | 5,798,880.30 | -0.0013282 | -0.01396178 |
|  | (110,468.90) | (3,312,889.00) | (0.0008926) | (0.01156410) |
| Month 35 ($\beta_{4}$)  (1-Month 35, 0-Other) | -763,502.88^**^ | -22,048,217.00^***^ | 0.0025763 | 0.0384377 |
|  | (299,158.20) | (7,930,205.00) | (0.0018762) | (0.0259121) |
| Month 35 $\times$ Time ($\beta_{5}$) | 385,524.84 | 13,466,353.00 | 0.00013134 | 0.00057308 |
|  | (255,507.20) | (7,208,067.00) | (0.0017986) | (0.0242306) |
| Site ($\beta_{6}$)  (1 Treatment, 0-Other) | -505,172.71^***^ | -242,4836.70 | -0.00064034 | -0.00453104 |
|  | (69,168.39) | (3,305,419.00) | (0.0015056) | (0.01425530) |
| Site$\times$Time ($\beta_{7}$) | -2,900.47 | -40,665.11 | 0.0000163 | 0.0002912 |
|  | (3,968.17) | (188,312.60) | (0.0000866) | (0.0008318) |
| Site$\times$Month 32 ($\beta_{8}$)  (1-Month 32, 0-Other) | 3,485,827.60^***^ | -1,489,067.30 | -0.00994859^***^ | -0.1091758^***^ |
|  | (210,421.30) | (6,953,844.00) | (0.00192840) | (0.0248992) |
| Site$\times$Month 32$\times$Time ($\beta_{9}$) | -744,684.97^***^ | 135,473.07 | 0.0052992^***^ | 0.0830776^***^ |
|  | (156,226.60) | (4,687,191.00) | (0.0013445) | (0.0163541) |
| Site$\times$Month 35 ($\beta_{10}$)  (1-Month 35, 0-Other) | -130,560.47 | 1,513,154.10 | -0.0072884^**^ | -0.1335265^***^ |
|  | (423,073.50) | (11,100,000.00) | (0.0028088) | (0.0366452) |
| Site$\times$Month 35$\times$Time ($\beta_{11}$) | 169,299.07 | -4,193,770.80 | -0.00287491 | -0.06007991 |
|  | (361,341.80) | (10,000,000.00) | (0.0026045) | (0.03426730) |
| $y_{t-1}$ ($\beta_{12}$) |  | 0.50743652^***^ | 0.5775596^***^ |  |
|  |  | (0.11365100) | (0.0969105) |  |
| No. of observations | 72 (36 per site) | 70 (35 per site) | 70 (35 per site) | 70 (35 per site) |
| R-square | 0.9684 | 0.5710 | 0.9572 | 0.4733 |
| Adjusted R-square | 0.9626 | 0.4806 | 0.9482 | 0.3734 |
| Overall Significance | $F_{\left( 11,60 \right)}=167.25$^***^ | $F_{\left( 12,57 \right)}= 6.32$^***^ | $F_{\left( 12,57 \right)}106.19$^***^ | $F_{\left( 11,58 \right)}= 4.74$^***^ |
| Rho | −0.135 | 0.2781698 | 0.6198382 | 0.4465161 |
| Durbin Watson (Autocorrelation) | $d_{\left( 12, 72 \right)}= 2.224$  inc^1^ (1%, 5%) | $d_{\left( 13, 70 \right)}= 1.668$  inc^1^ (1%, 5%) | $d_{\left( 13,70 \right)}= 1.012$  pos^2^ (1%, 5%) | $d_{\left( 12,70 \right)}= 1.117$  inc^1^ (1%), pos^2^ (5%) |
| Durbin Watson Transformed | $d_{\left( 7, 72 \right)}= 2.026$  none (1%), inc^1^ (5%) | $d_{\left( 7, 72 \right)}= 1.935$  none (1%), inc^1^ (5%) | $d_{\left( 7,72 \right)}= 1.806$  inc^1^ (1%, 5%) | $d_{\left( 12,70 \right)}= 1.956$  none (1%), inc^1^ (5%) |
| Legend: *** (Significant at 1% & 5%); **(Significant at 5% only);  Notes   1. inc – shortened for inconclusive. 2. pos – shortened for positive 3. The proportion change is: $proportion change= \frac{{SugarContent}_{t}- {SugarContent}_{t-1}}{{SugarContent}_{t-1}}$ where t is the current month, and t-1 is the previous month | | | | |
