# Supplementary material for: Evaluating the impact of marketing interventions on sugar-free and sugar-sweetened soft drink sales and sugar purchases in a fast-food restaurant setting
Source: BMC Public Health. 2023 Aug 18;23:1578. doi: 10.1186/s12889-023-16395-z (PMC10439673; doi:10.1186/s12889-023-16395-z)
Supplement: Supplementary file 2 — Additional file 2: Table A2. Calculated combined coefficients from the results: treatment and control sites. [file 12889_2023_16395_MOESM2_ESM.docx]

**Table A2 – Calculated combined coefficients from the results: treatment and control sites**

|  | Dependent Variable ($y_{t}$) | | | |
| --- | --- | --- | --- | --- |
|  | Sugar-free drinks (Total ml) | Sugar-sweetened  drinks  (Total ml) | Sugar purchased (Grams per ml) | Proportion Change^1^  Sugar purchased |
| Intercept ($\beta_{0}$) | 1,712,962.700 | 20,701,560.000 | 0.03613533 | 0.00528150 |
| Time  ($\beta_{1}$) | 14,469.358 | -35,075.310 | 0.00005548 | -0.00015723 |
| Month 32  ($\beta_{2}$) | 1,012,38.330 | -3,629,065.600 | 0.00054436 | 0.00490275 |
| Month 32 $\times$ Time  ($\beta_{1}+\beta_{3}$) | 257,369.888 | 5,763,804.99 | -0.00127272 | -0.01411898 |
| Month 35  ($\beta_{4}$) | -763,502.880 | -22,048,217.000 | 0.00257630 | 0.03843770 |
| Month 35 $\times$ Time  (${\beta_{1}+\beta_{3}+\beta}_{5}$) | 642,894.728 | 19,230,157.99 | -0.00114138 | -0.0135459 |
| Site  (${\beta_{0}+\beta}_{6}$) | 1,207,790.290 | 18,276,723.300 | 0.03549499 | 0.00075010 |
| Site$\times$Time  ($\beta_{1}+\beta_{7}$) | 11,568.890 | -75,740.424 | 0.00007175 | 0.00013400 |
| Site$\times$Month 32  ($\beta_{2}+\beta_{8}$) | 3,587,065.930 | -5,118,132.900 | -0.00940423 | -0.10427305 |
| Site$\times$Month 32$\times$Time  ($\left[ \beta_{1}+\beta_{7} \right]+[\beta_{3}+ \beta_{9}]$) | -501,784.440 | 5,858,612.946 | 0.00404278 | 0.06924982 |
| Site$\times$Month 35  ($\beta_{4}+\beta_{10}$) | -894,063.350 | -20,535,062.900 | -0.00471205 | -0.09508888 |
| Site$\times$Month 35$\times$Time  ($\left[ \beta_{1}+\beta_{7} \right]+\left[ \beta_{3}+ \beta_{9} \right]+[{\beta_{5}+\beta}_{11}]$) | -216,225.770 | 15,131,195.146 | 0.00129921 | 0.009742990 |
| $y_{t-1}$ ($\beta_{12}$) |  | 0.5074365200 | 0.5775596 |  |
| Notes   1. The proportion change is: $proportion change= \frac{{SugarContent}_{t}- {SugarContent}_{t-1}}{{SugarContent}_{t-1}}$ where t is the current month, and t-1 is the previous month | | | | |
