# Supplementary material for: Evaluating the impact of marketing interventions on sugar-free and sugar-sweetened soft drink sales and sugar purchases in a fast-food restaurant setting
Source: BMC Public Health. 2023 Aug 18;23:1578. doi: 10.1186/s12889-023-16395-z (PMC10439673; doi:10.1186/s12889-023-16395-z)
Supplement: Supplementary file 5 — Additional file 5: Explanation for Single Site Treatment Results. [file 12889_2023_16395_MOESM5_ESM.docx]

Appendix 1

## Single-group analysis

The interrupted time series analysis results for the treatment site are in Supplementary Tables C1 and C2, and the relevant graphs are in Supplementary Figures C1 to C4. The base equation for the single-group analysis is below.

$$Y_{t}= \beta_{0}+ \beta_{1}T_{t}+ \beta_{2}I_{1_{t}}+ \beta_{3}I_{1_{t}}T_{t}+ \beta_{4}I_{2_{t}}+ \beta_{5}I_{2_{t}}T_{t}+\epsilon_{t}$$

The equation above was estimated separately for volume sales of sugar-free drinks, sugar-sweetened drinks, the sugar purchased of all soft drinks sold, and monthly proportion change in sugar purchased. These are represented in the dependent variable $Y_{t}$. It is modelled based on the initial level ($\beta_{0}$), time ($T_{t}$), the introduction of the intervention ($I_{1_{t}}$ an $I_{2_{t}}$) and after the introduction ($I_{1_{t}}T_{t}$ and$I_{2_{t}}T_{t}$).

As the analysis uses time blocks, the equation's relevant components will differ between phases as below:

1. **Pre-intervention** – This phase is represented by the periods before the vertical dotted line in Supplementary Figure 1, i.e. months 1-31. The operational components before the intervention include the initial level ($\beta_{0}$) and the periods designated as $T_{t}$. The rest of the equation will not apply as no intervention exists during this phase ($I_{t}=0$). Thus, the coefficient $\beta_{1}$ will show the average change in sales volume or sugar purchased before the intervention.
2. **Introduction phases** – The first vertical line in Supplementary Figure 1 shows the period when all five interventions were first introduced in Month 32 (see also Supplementary Table C1). The coefficient, $\beta_{2}$ captures the initial change in sales or sugar purchased at introduction. The second vertical line shows the period where only interventions 4 and 5 were active in month 35. The change is captured by the coefficient $\beta_{4}$. The double-headed arrows indicate the magnitude of these changes.
3. **Post-introduction phases** – This is the period after the vertical dotted lines in Supplementary Figure C1. The coefficient, $\beta_{3}$ captures the change in sales or sugar purchased after the introduction of all five interventions in Month 32. The changes after Month 35 are captured by $\beta_{5}$.

**Supplementary Figure C1 - Phases of the single group interrupted time series analysis**

Adapted from Linden [33]

INSERT SUPPLEMENTARY FIGURE C1 HERE

The summary of results for different types of soft drinks when comparing pre-intervention and post-intervention stages are shown in Supplementary Table C1 and summarised below:

## Impact on sales of sugar-free drinks (SFDs)

The introduction of the interventions had the most significant impact on SFD sales. Supplementary Figure C2 shows the changes in the slopes for the predicted volume sales at the different phases. The fitted line at pre-intervention is much flatter than at post-introduction ( Months 32 and 35), indicating that volume sales changed gradually from months 1-31 and more dramatically in month 32.

**Supplementary Figure C2 - Volume sales of sugar-free drinks: treatment site**

INSERT SUPPLEMENTARY FIGURE C2 HERE

The estimated model results in Supplementary Table C1 confirmed that the differences between the three phases are significant. All the coefficients are statistically significant at 1% in phases 1 and 2 and 5% in phase 3. The estimated model explains 98.50% (Adj R-Square) of the variation in volume sales. The different stages collectively explain the volume sales of sugar-free drinks as indicated by the overall significance level.

Supplementary Table C1 (column 2) shows that SFD sales in months 1-31 were already increasing but only at an average of 0.82 % per month. In month 32, when all five interventions were introduced, SFD sales increased sharply by 230.50 % from month 31. In months 33-34, when intervention 1 (FC) was discontinued, sales decreased by an average of 9.66% per month. Volume sales further dropped by 36.97% in month 35 when interventions 2 and 3 (POP, PR1) were withdrawn. It then increased by 9.19% afterwards. SFD sales during months 32-35 were, on average, 147.42% higher per month than in the pre-intervention period.

## Impact on sales of sugar-sweetened drinks (SSDs)

The interventions had a lesser impact on SSD sales. The distance between the fitted lines before and after introduction in month 32 in Supplementary Figure C3 is negligible. In month 32, the SSD sales decreased by 10.91% from the previous month, but it was not statistically significant (see Supplementary Table C1).

**Supplementary Figure C3 - Volume sales of sugar-sweetened drinks: treatment site**

INSERT SUPPLEMENTARY FIGURE C3 HERE

In months 33-34, when intervention 1 (FC) was withdrawn, SSD sales increased by an average of 18.76% per month. The sharp drop of 41.78% in month 35 was statistically significant at 1%, as interventions 2 and 3 (POP, PR1) were discontinued. It should be noted that during this same period, SFD sales also declined. In month 36, although SSD sales rose by 14.50%, it was not statistically significant.

The estimated model (Supplementary Table C1) shows an adjusted R square of 48.44. Despite accounting for the effects of the previous month’s sales, the autocorrelation was not completely corrected. The adjustments only led to no autocorrelation at 1% and was inconclusive at 5%.

## Impact on sugar purchased

The rise in SFD sales in months 32 -35 led to an increase in total soft drinks sales and a reduction in in-store sugar purchases. As shown in Supplementary Figure C4, the amount of sugar purchased during the pre-intervention and post-introduction phases is notably different. In month 32, when all interventions were present, sugar purchased fell by 10.75% (*P* < .01) from month 31(end of preintervention), but when intervention 1 was withdrawn in months 33-34, it dropped momentarily before rising again. The average change of 1.22% per month is statistically significant at 1%. In month 35, when interventions 2 and 3 (POP, PR1) were withdrawn, it fell by only 3.62% but was significant at 5%. Then in month 36, although it rose by 2.16%, this was not statistically significant.

**Supplementary Figure C4 - Sugar purchases: treatment site**

INSERT SUPPLEMENTARY FIGURE C4 HERE

Although the model explained 93.33% (Adj R square) of the variation in sugar purchased, it cannot be determined whether positive autocorrelation was completely addressed, as there was no autocorrelation at 1% and was inconclusive at 5% despite the adjustments. A proportion change in sugar purchased model was then estimated.

## Proportion change in sugar purchases

The proportion change (in absolute values) from the previous month was used to address possible autocorrelation in the sugar purchased estimates. In months 1-31, the proportion change in sugar purchased increased steadily at 0.01340% per month, but in month 32, when all five interventions were introduced, it dropped by 10.43% (*P* <.01) from the previous month. In months 33-34, when intervention 1 (FC) was withdrawn, sugar purchased rose by 6.91% per month, and in month 35, when interventions 2 & 3 (POP, PR1) were withdrawn, it dropped by 9.5% ( *P* <.01) from the previous month. The relatively larger drop in SSD sales had caused sugar purchases to also fall in month 35.

**Supplementary Figure C5- Monthly proportion change in sugar purchases: treatment site**

INSERT SUPPLEMENTARY FIGURE C5 HERE

The estimated proportion change model, as shown in Supplementary Table C1, explains 56.26% of the variation in the changes in sugar purchased between months. This is considered a better model because there was no autocorrelation at 1% and 5%.

**Supplementary Table C1 – Results of the interrupted time series analysis: treatment site**

INSERT SUPPLEMENTARY TABLE C1 HERE

In summary, Supplementary Table C1 shows that the interventions had a statistically significant effect on the volume sales of sugar-free soft drinks and less on SSD. The interventions had an impact on sales of SSD only in months 33-35, while its effects on SFT sales were significant throughout months 31-36.

In this study, a desirable outcome would be that the interventions increased SFD sales and decreased SSD. Sugar purchased overall was lower in the experimental than in the pre-intervention period (see Fig 4).

The linear trend in post introduction in Supplementary Table C2 further reinforces the observation that had the number of interventions remained constant, their impact on sugar purchased and proportion change could have been statistically significant in months 35 -36. Had the number of interventions not been reduced to 2 in month 35, SSD could have had a smaller or negative coefficient, i.e. a smaller increase or a decrease in sales.

**Supplementary Table C2 – Linear trend post-introduction: treatment site**

INSERT SUPPLEMENTARY TABLE C2 HERE
