# Supplementary figures and images for: Evaluating the impact of marketing interventions on sugar-free and sugar-sweetened soft drink sales and sugar purchases in a fast-food restaurant setting
Source: BMC Public Health. 2023 Aug 18;23:1578. doi: 10.1186/s12889-023-16395-z (PMC10439673; doi:10.1186/s12889-023-16395-z)

**Figure C1 - Phases of the single group interrupted time series analysis**

Adapted from Linden [33]


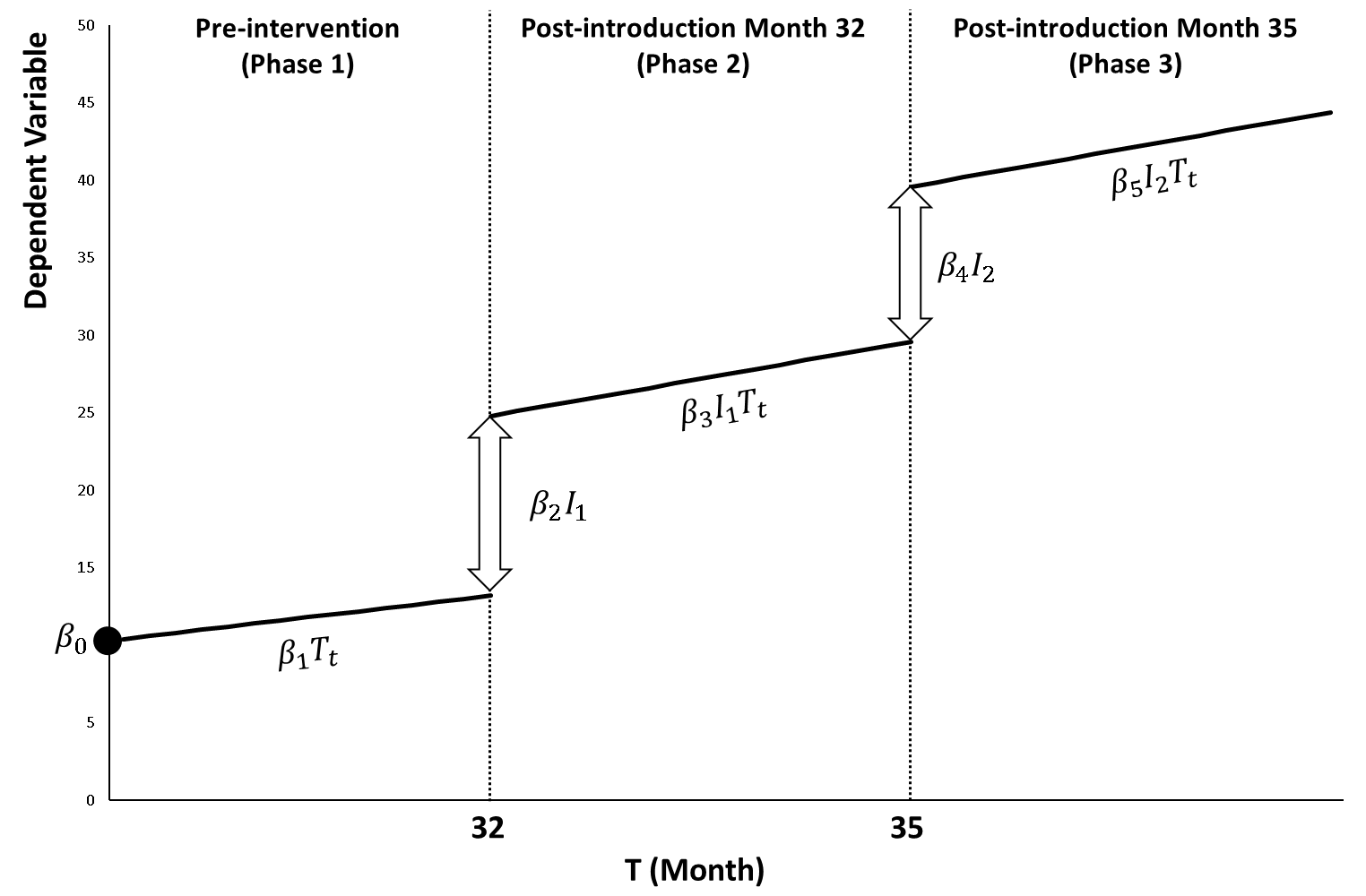

Supplement: Supplementary file 6 — Additional file 6: Figure C1. Phases of the single group interrupted time series analysis. [file 12889_2023_16395_MOESM6_ESM.docx]
