# Supplementary material for: Evaluating the impact of marketing interventions on sugar-free and sugar-sweetened soft drink sales and sugar purchases in a fast-food restaurant setting
Source: BMC Public Health. 2023 Aug 18;23:1578. doi: 10.1186/s12889-023-16395-z (PMC10439673; doi:10.1186/s12889-023-16395-z)
Supplement: Supplementary file 8 — Additional file 8: Figure C3. Volume sales of sugar-sweetened drinks: treatment site. [file 12889_2023_16395_MOESM8_ESM.docx]

**Figure C3 - Volume sales of sugar-sweetened drinks: treatment site**
