# Supplementary material for: Evaluating the impact of marketing interventions on sugar-free and sugar-sweetened soft drink sales and sugar purchases in a fast-food restaurant setting
Source: BMC Public Health. 2023 Aug 18;23:1578. doi: 10.1186/s12889-023-16395-z (PMC10439673; doi:10.1186/s12889-023-16395-z)
Supplement: Supplementary file 11 — Additional file 11: Table C1. Results of the interrupted time series analysis: treatment site. [file 12889_2023_16395_MOESM11_ESM.docx]

**Table C1 - Results of the interrupted time series analysis: treatment site**

|  | Dependent Variable ($y_{t}$) | | | |
| --- | --- | --- | --- | --- |
|  | Sugar-free drinks (Total ml) | Sugar-sweetened drinks  (Total ml) | Sugar purchased  (Grams per ml) | Proportion Change^2^  Sugar purchased |
| Intercept | 1,209,175.00^***^ | 16,707,258.00^***^ | 0.03827208^***^ | 0.00075023 |
|  | (41,089.95) | (6,468,939.00) | (0.00213350) | (0.01009760) |
| Month  (1 to 36) | 11,392.03^***^ | -73892.394 | 0.00008062 | 0.00013398 |
|  | (2,362.06) | (127,393.30) | (0.00006980) | (0.00058920) |
| Month 32  (1-Month 32, 0-Else) | 3,601,243.00^***^ | 4,885,819.20 | -0.00946271^***^ | -0.1042732^***^ |
|  | (139,565.80) | (4,132,805.00) | (0.00134650) | (0.0176353) |
| Month 32 $\times$ Time | -474,958.30^***^ | 6,031,409.90^**^ | 0.00378403^***^ | 0.06911575^***^ |
|  | (51,952.21) | (2,758,932.00) | (0.00109590) | (0.0115830) |
| Month 35  (1-Month 35, 0-Else) | -1,102,714.00^***^ | -21,264,902.00^***^ | -0.0044196^**^ | -0.09508841^***^ |
|  | (333,617.30) | (6,605943.00) | (0.0020726) | (0.02595390) |
| Month 35 $\times$ Time | 709,054.60^**^ | 9,635,124.30 | -0.00251986 | -0.05950657^**^ |
|  | (288,493.50) | (5,938,156.00) | (0.00191790) | (0.0242700) |
| $y_{t-1}$ |  | 0.5513531^***^ | 0.54367798^***^ |  |
|  |  | (0.1623441) | (0.1279581) |  |
| No. of observations | 36 | 35 | 35 | 35 |
| R-square | 0.9872 | 0.5754 | 0.9390 | 0.6269 |
| Adjusted R-square | 0.9850 | 0.4844 | 0.9333 | 0.5626 |
| Overall significance | $F_{\left( 5,30 \right)}= 461.66$^***^ | $F_{\left( 6,28 \right)}= 6.32$^***^ | $F_{\left( 6,28 \right)}= 117.39$^***^ | $F_{\left( 5,29 \right)}= 9.75$^***^ |
| Rho | -0.4192946 | 0.3731792 | 0.670147 | 0.4465839 |
| Original Durbin Watson  (Autocorrelation) | $d_{\left( 6, 36 \right)}= 2.646$  inc^1^ (1% & 5%) | $d_{\left( 7,35 \right)}= 1.488$  inc^1^ (1% & 5%) | $d_{\left( 7, 35 \right)}= 0.981$  inc^1^ (1%), positive (5%) | $d_{\left( 6, 35 \right)}= 1.105$  inc^1^(1% & 5%) |
| Transformed Durbin Watson  (Autocorrelation) | $d_{\left( 6, 36 \right)}= 1.925$  none (1% & 5%) | $d_{\left( 7,35 \right)}= 1.919$  none (1%), inc^1^ (5%) | $d_{\left( 7, 35 \right)}= 1.771$  None (1%), inc^1^ (5%) | $d_{\left( 6, 35 \right)}= 1.984$  none (1% & 5%) |
| Legend: *** (Significant at 1% & 5%); **(Significant at 5% only)  Notes   1. inc – shorterend for inconclusive 2. The proportion change is: $proportion change= \frac{{SugarContent}_{t}- {SugarContent}_{t-1}}{{SugarContent}_{t-1}}$ | | | | |
