# Supplementary material for: Evaluating the impact of marketing interventions on sugar-free and sugar-sweetened soft drink sales and sugar purchases in a fast-food restaurant setting
Source: BMC Public Health. 2023 Aug 18;23:1578. doi: 10.1186/s12889-023-16395-z (PMC10439673; doi:10.1186/s12889-023-16395-z)
Supplement: Supplementary file 12 — Additional file 12: Table C2. Linear trend post-introduction: treatment site. [file 12889_2023_16395_MOESM12_ESM.docx]

**Table C2 - Linear trend post-introduction: treatment site**

|  | Sugar-free drinks (Total ml) | Sugar-sweetened drinks  (Total ml) | Sugar purchased  (Grams per ml) | Proportion Change^1^  Sugar purchased |
| --- | --- | --- | --- | --- |
| Month 32 | −464,000.00^**^ | 5960000.00^**^ | 0.0039^***^ | 0.0692^***^ |
|  | (111,000.00) | (2740000.00) | (0.0011) | (0.0115) |
| Month 35 | −245,000.00^***^ | 15600000.00^***^ | 0.0013 | 0.0097 |
|  | (293,000.00) | (5030000.00) | (0.0014) | (0.0197) |
| Legend: *** (Significant at 1%, 5% & 10%); **(Significant at 5% & 10% only); * (Significant at 10% only)  Notes   1. The proportion change is: $proportion change= \frac{{SugarContent}_{t}- {SugarContent}_{t-1}}{{SugarContent}_{t-1}}$ | | | | |
